# Is there a role for HSF1 in viral infections?

**DOI:** 10.1002/2211-5463.13419

**Published:** 2022-05-10

**Authors:** Antonia Reyes, Areli J. Navarro, Benjamín Diethelm‐Varela, Alexis M. Kalergis, Pablo A. González

**Affiliations:** ^1^ Millennium Institute on Immunology and Immunotherapy Departamento de Genética Molecular y Microbiología Facultad de Ciencias Biológicas Pontificia Universidad Católica de Chile Santiago Chile; ^2^ Departamento de Endocrinología Facultad de Medicina Escuela de Medicina Pontificia Universidad Católica de Chile Santiago Chile

**Keywords:** heat shock, heat shock factor 1, HSF1, stress, viral infections

## Abstract

Cells undergo numerous processes to adapt to new challenging conditions and stressors. Heat stress is regulated by a family of heat shock factors (HSFs) that initiate a heat shock response by upregulating the expression of heat shock proteins (HSPs) intended to counteract cellular damage elicited by increased environmental temperature. Heat shock factor 1 (HSF1) is known as the master regulator of the heat shock response and upon its activation induces the transcription of genes that encode for molecular chaperones, such as HSP40, HSP70, and HSP90. Importantly, an accumulating body of studies relates HSF1 with viral infections; the induction of fever during viral infection may activate HSF1 and trigger a consequent heat shock response. Here, we review the role of HSF1 in different viral infections and its impact on the health outcome for the host. Studying the relationship between HSF1 and viruses could open new potential therapeutic strategies given the availability of drugs that regulate the activation of this transcription factor.

## Graphical abstract

Heat shock factor 1 (HSF1) plays important roles in the replication cycle of viruses. Numerous viruses have been found to modulate, or to be affected by the activation of HSF1. Depending on the virus, HSF1 can lead to detrimental effects over the virus (red line) or confer advantages to the virus (green arrows). Understanding the interplay between HSF1 and viral infections could help identify novel pharmacological targets for the development of antiviral therapeutic approaches.

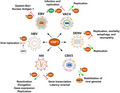

**Abbreviations**
AIDS: acquired immunodeficiency syndrome
AKT: Akt serine/threonine kinase
ARE: AU‐rich element
Atg7: autophagy‐related protein 7
CaMKIIγ: Ca2+/calmodulin‐dependent protein kinase γ
CCT: chaperonin containing tailless complex polypeptide
CVB3: coxsackievirus B3
DBD: DNA‐binding domain
DENV: dengue virus
EBNA1: Epstein–Barr nuclear antigen 1
ERK1: extracellular signal‐regulated kinase 1
GSK3: glycogen synthase kinase 3
HBV: hepatitis B virus
HCMV: human cytomegalovirus
HIV: human immunodeficiency virus
Hop: heat‐shock organizing protein
HR‐A/B: heptad repeat regions A and B
HSE: heat shock response elements
HSF1: Heat shock factor 1
HSFs: Heat shock factors
HSP70: heat shock protein of 70 kDa
HSP90: heat shock protein of 90 kDa
HSPs: Heat shock proteins
HSV‐1: herpes simplex virus 1
HSV‐2: herpes simplex virus 2
IRES: internal ribosome entry site
LRAs: latency‐reversing agents
LTR: long terminal repeat
p38 MAP: p38 mitogen‐activated protein kinase
PI3K: phosphatidylinositol 3‐kinase
TRiC: tailless complex polypeptide 1 ring complex
UPR: unfolded protein response
VACV: vaccinia virus

Heat shock factors (HSFs) are a family of transcription factors that are mostly activated in response to cell stress induced by heat, with heat shock factor 1 (HSF1) being the most studied component of this family [1, 2, 3]. HSF1 and other HSFs can bind to specific regions in the genome named heat shock response elements (HSE), which have known consensus sequences [4, 5]. HSF1 is conserved from fungi to vertebrates and regulates the transcription of multiple genes, most of them oriented at easing cell damage elicited by heat stress [3, 6]. HSFs can induce the transcription of a set of genes that encode proteins involved in the heat shock response (HSR), such as chaperones and heat shock proteins (HSPs), which play numerous roles in controlling cell deregulation produced by elevated environmental temperatures. More recently, the unfolded protein response (UPR) has also been reported to be related to heat shock stress components, such as HSF1 and HSP47 [7, 8].

Seven members of the HSF family have been identified in eukaryotes: HSF1‐HSF5, HSFY, and HSFX [9, 10]. At present, relatively little is known regarding the roles of HSF5, HSFY, and HSFX [11]. On the contrary, HSF1‐HSF4 have been isolated in vertebrates and more extensively studied, as well as the single HSF gene known to date to be encoded in yeast [5, 12]. Additionally, HSF1, HSF2, and HSF4 are expressed as different isoforms [5, 12, 13]. HSF2α and HSF2β are expressed in vertebrates, and HSF2 has been reported to act as a transcriptional regulator for the HSF1‐dependent activation of HSP genes [14, 15]. Additionally, it has been shown that HSF2 is not activated by heat shock, but nevertheless colocalizes and interacts with HSF1 [16, 17]. Interestingly, neither HSF4 nor its two HSF4 isoforms, namely HSF4a and HSF4b, are activated by heat shock [12, 18]. In fact, it has been reported that these HSF4 isoforms have opposing effects on the basal levels of chaperone gene expression, with HSF4a attenuating the expression of these genes and consequently the induction of HSPs, likely due to a direct competition with HSF1 at binding to HSEs, while HSF4b induces the transcription of heat shock response genes [12, 19].

Four isoforms of HSF1 have been reported to date: HSF1α, HSF1β, HSF1γα, and HSF1γβ [10, 20]. HSF1 is formed by an N‐terminal winged helix‐turn‐helix DNA‐binding domain and hydrophobic heptad repeat regions A and B (HR‐A/B), which are thought to act as a leucine zipper coiled‐coil trimerization domain [21, 22, 23, 24, 25]. There is also a regulatory domain, HR‐C and a C‐terminal transcription activation domain [21, 23, 24, 25]. Under non‐stress conditions, HSF1 is in a monomeric form and is associated as a complex with molecular chaperones, such as the HSP of 70 kDa (HSP70), or the HSP of 90 kDa (HSP90), and it is also regulated by the tailless complex polypeptide 1 ring complex (TRiC), also known as the chaperonin containing tailless complex polypeptide 1 (CCT) [26, 27, 28, 29, 30].

Heat shock factor 1 strongly participates in response to heat shock by inducing the expression of HSR genes, such as molecular chaperones or HSPs [1, 6, 31]. These proteins play major roles in the HSR by promoting cell survival [1, 6]. However, the role of HSF1 is much wider, involving functions beyond the HSR [6]. Over the last decades, this transcription factor has been reported to participate in multiple cellular processes, such as apoptosis, the unfolded protein response (UPR) in the endoplasmic reticulum, oxidative stress, autophagy, multidrug resistance, and physiological development, among others [3, 6, 8].

Additionally, a role between HSF1 and viral infections has been described, although in a somewhat limited manner despite the fact that HSPs are known to participate in many processes related to viral infection, such as viral entry, viral replication, and viral gene expression, among others [32]. Furthermore, it is unknown if HSF1 may have a potential as a new therapeutic target for different viral infections. Given that new drugs that block or activate HSF1 are currently being tested in clinical trials, we sought studies that relate HSF1 with viral infection and found that because this transcription factor participates in the replication cycle of many viruses, its modulation could eventually exert a favorable influence over the host’s ability to control or resolve viral infections [33, 34, 35]. Here, we review aspects related to HSF1 activation and discuss what is known regarding the role of HSF1 during viral infections.

## HSF1 activation

Heat stress, which may be considered as temperatures between 39 and 43 °C, the presence of heavy metals, oxidants, or proteotoxic agents induce the homotrimerization of HSF1, its dissociation from chaperones, and its phosphorylation which leads to its active form [28, 36, 37, 38]. Because fever frequently occurs upon viral infections, HSF1 activation may be common during viral infections, although this has not been studied extensively [36, 39].

In its homotrimer form, HSF1 translocates to the nucleus, which leads to its accumulation in this compartment [40, 41]. Here, it can bind to specific DNA sequences in the genome named heat shock elements (HSE), which are usually located in the upstream untranslated region of target genes [1, 6, 42]. HSE are composed of a small pentameric consensus sequence containing the xGAAx motif [42]. However, a stable association between HSF1 and DNA requires three overlapped pentameric sequences with the following composition TTCxxGAAxxTTC [4]. Once HSF1 binds to the DNA, it will upregulate the transcription of the genes encoding this element, many of which are HSPs [1, 3, 32].

Interestingly, the activation of this transcription factor is mediated by both protein–protein interactions and post‐translational modifications which are discussed below in the following paragraphs [21, 38]. Furthermore, different studies suggest that the activation cycle of this transcription factor is highly regulated [21, 24, 25, 38]. Indeed, it is thought that HSF1 is activated by different mechanisms based on the type of stress, with its activation depending on whether the stimulus is thermal or non‐thermal [3].

Multiple factors are believed to be involved in the activation of HSF1. For instance, it has been reported that temperature increases cause intrinsic structural changes in HSF1 that support its oligomerization and activation [43]. Temperature‐induced conformational dynamics of HSF1 revealed that at 20 °C only few regions of this protein, such as the DNA‐binding domain (DBD), the oligomerization domain (LZ1‐3) and HR‐C are structured [3, 43]. Furthermore, it has been reported that at higher temperatures there is a temperature‐dependent unfolding process of HR‐C, which is known to repress HSF1 trimerization via a coiled‐coil interaction with HR‐A, or HR‐B in non‐stressed cells [24, 43]. The unfolding of HR‐C leads to the stabilization of HR‐A/B, which is known as the trimerization domain [24, 43]. This finding indicates that HSF1 has an intrinsic capacity to sense temperature changes. Interestingly, the temperature at which HSF1 is activated has been shown to be tissue‐dependent [44]. Additionally, differential temperature sensitivities have been observed for this transcription factor in different organisms, even with identical primary protein sequences [44]. This finding suggests that it is likely that cell‐specific protein–protein interactions with HSF1 can also modulate the structure of this protein and alter the thresholds required for its stabilization involved in its activation.

Importantly, there is a negative autoregulatory feedback loop, which guarantees that HSF1 HSRs occur at a level consistent with the extent of the protein damage in the cell [30, 45]. For instance, some HSPs induced by HSF1, such as HSP70, HSP72, and HSP90, can inhibit HSF1 by impairing the formation of new active HSF1 trimers, by directly binding to the trimerization domain of this factor [6, 30]. Additionally, in nematodes and mammals it has been reported that the activity of HSF1 can be elicited by stress‐induced kinases. Indeed, glycogen synthase kinase 3 (GSK3), extracellular signal‐regulated kinase 1 (ERK1), and p38 mitogen‐activated protein kinase (p38 MAP) are all able to inhibit HSF1 activity, while Akt serine/threonine kinase (AKT), phosphatidylinositol 3‐kinase (PI3K), and cAMP‐dependent protein kinase A promote the activity of HSF1 [46, 47, 48]. Importantly, numerous viruses are known to modulate some of these factors, such as the human cytomegalovirus (HCMV) and herpes simplex viruses 1 and 2 (HSV‐1 and HSV‐2), which are known to temporally and differentially regulate the PI3K/Akt pathway during infection [49, 50]. Moreover, it has been shown that HCMV can induce the mTOR pathway during cellular stress and that this pathway is important during lytic infections [49]. It has also been reported that infection with the human immunodeficiency virus (HIV) can be repressed by downregulating ERK 1/2 and p38 MAPK [51]. Additionally, it has been reported that the mTOR pathway is increased in HIV infections, which in turn promotes viral integration and replication [52]. Another study showed that HIV infection inhibits Akt phosphorylation and the PI3K/Akt signaling pathway in plasmacytoid dendritic cells [53]. The hepatitis B virus (HBV) protein x (HBx) activates the ERK and p38MAPK signaling pathways, which in turn promote metastasis of liver cancer [54].

**Fig. 1:**
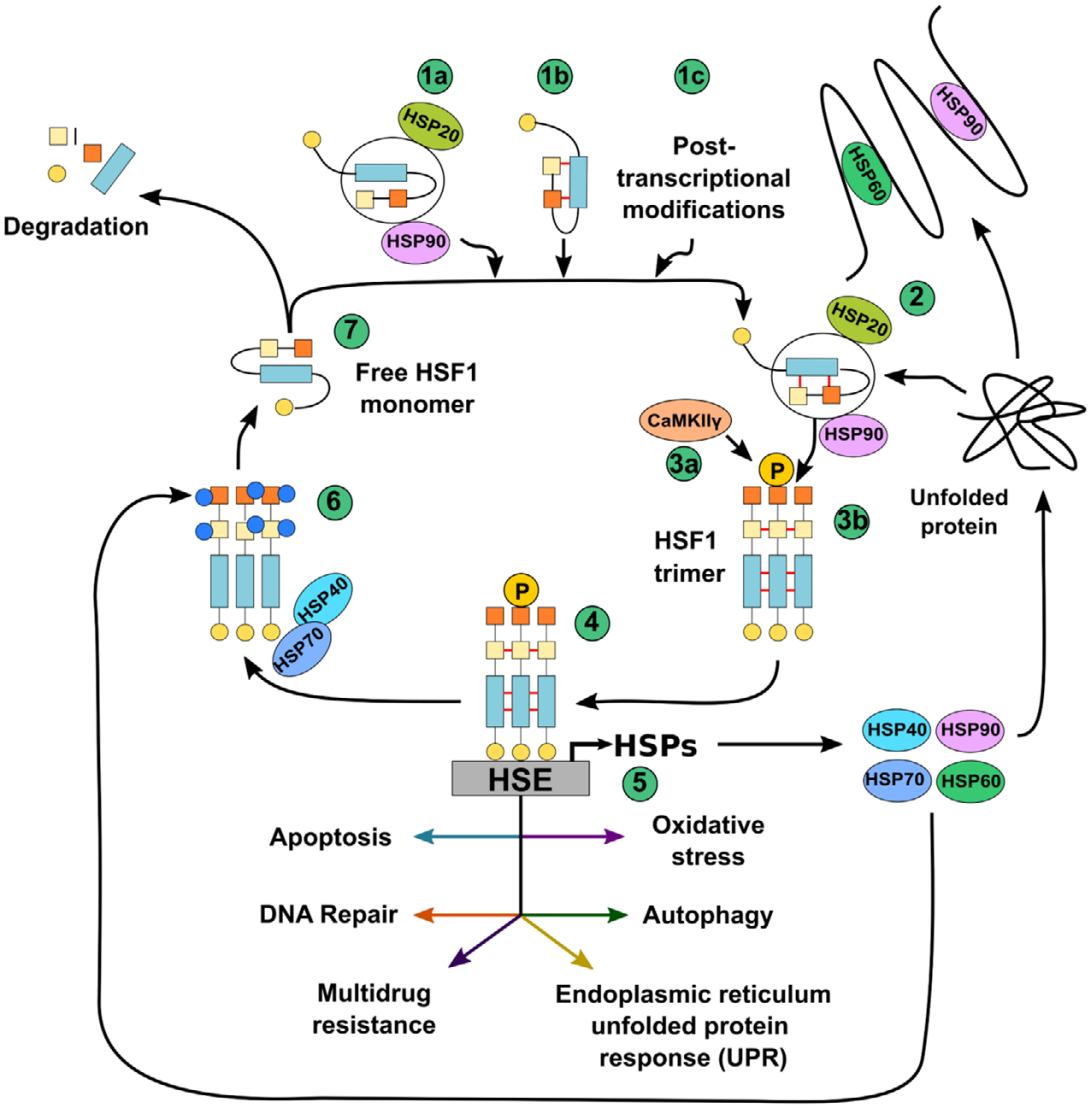
HSF1 activation. HSF1 is usually present in the cell in an inactivated form. Inactivation of HSF1 occurs mainly by three mechanisms: (1a) HSP90 binding to HSF1, (1b) HSF1 stabilization through the formation of a leucine zipper structure within the protein (red lines), or (1c) through post‐transcriptional modifications, such as acetylation, sumoylation, and phosphorylation. (2) HSF1 is activated when an increase in misfolded proteins occurs in the cell, such as after heat shock (increased environmental temperature). HSF1 activation involves the release of monomeric HSF1 from chaperones, such as HSP20 and HSP90 (3a). Once activated, HSF1 monomers interact together to form a trimer that is stabilized by leucine zippers (red lines) (3b) and is phosphorylated by the calcium/calmodulin‐dependent protein kinase II gamma (CaMKIIγ). (4) HSPs act as molecular chaperones for the correct folding of numerous proteins in the cell. (5) HSF1 binds to DNA sequences in the genome, namely heat shock elements (HSE) in the promoters of genes encoding for heat shock responses, such as heat shock proteins (HSPs) promoting their transcription. HSF1 also promotes the transcription of genes involved in the regulation of apoptosis, DNA repair, modulation of drug resistance, unfolded protein response (UPR) at the endoplasmic reticulum, autophagy, and oxidative stress, among others. (6) Acetylation (blue circles) of HSF1 at Lys80 destabilizes its interaction with the DNA. HSP40 together with HSP70 bind to specific sites in HSF1 monomers leading to a destabilization of the trimer. (7) Excess HSF1 is degraded through the SCFβ‐TrCP pathway, and only a basal amount of inactive HSF1 remains in the cell.

It is also important to mention that post‐transcriptional modifications can also modulate HSF1 activation. For instance, acetylation of lysine residues Lys208 and Lys298 in HSF1 leads to protein stabilization [55, 56]. In contrast, acetylation of Lys80 and Lys118 leads to the inhibition of HSF1, similar to the effect induced by sumoylation of Lys298 [56, 57, 58, 59]. Thus, it is possible to envisage that viral infections could modulate the action of enzymes that modify HSF1 in a post‐translational manner, in such a way to favor or inhibit its activation. An overall representation of HSF1 activation is depicted in Fig. 1.

## HSF1 and viral infections

Although HSPs play important roles in the replication cycle of many viruses, as extensively reviewed by Wan et al. [32], the role of HSF1 in viral infections has been scarcely described. This is particularly surprising, given that a sudden increase in body temperature, such as might occur with fever is a frequent host response during viral infections, and thus, HSF1 may be activated under these circumstances and have an impact on the progression of the infections [60, 61]. Consequently, its modulation may impact the host–pathogen interaction [38, 39, 62].

At present, most studies evaluating a role for HSF1 in viral infections are focused on the activation of this transcription factor during infection caused by the human immunodeficiency virus (HIV). To date, HSF1 has been reported to participate in the transcription of HIV genes and the reactivation of this virus from latency [62]. HSF1 has been found to bind to the 5′‐LTR of HIV, which leads to viral reactivation and the recruitment of multi‐subunit complexes, such as p300 which is recruited by HSF1 for self‐acetylation and p‐TEFb that is involved in the regulation of transcription [62]. Importantly, knocking out the gene encoding for HSF1 in 293T cells (293T‐HSF1‐KO; −4/−10 bp) led to impaired transcription of viral genes [62]. Conversely, the overexpression of HSF1 increased the transcription of HIV genes [62]. Thus, HSF1 positively regulates the transcription of latent HIV. Additionally, HSF1 has been described to be constitutively active in T cells latently infected with HIV and to likely contribute to viral reactivation. Therefore, HSF1‐mediated HIV reactivation may occur in response to stress factors over these cells, such as starvation or increased temperature due to fever [62].

Furthermore, *in vitro* studies have shown that the mechanism of action of some latency‐reversing agents (LRAs) involves a HSF1‐mediated stress pathway [63]. Consequently, the inhibition of HSF1 decreased latency reversal, and thus negatively modulating this factor holds the potential to delay the acquired immunodeficiency syndrome (AIDS) [63]. Additionally, HSF1 stimulates HIV elongation via the recruitment of p‐TEFb to the viral long terminal repeat (LTR), and the inhibition of HSF1 dampens the formation of elongated HIV‐1 transcripts [63]. Moreover, Nef (an HIV protein)‐dependent induction of HSP40 has been reported to lead to increased HIV gene expression [64], and HSF1 binds directly to a HSF1‐binding sequence in the HIV LTR promoter, which leads to an increase in viral gene expression and replication [64]. These findings suggest that HSF1‐mediated signaling plays a role in HIV‐1 transcriptional elongation.

However, stress‐independent activation of HSF1 can reduce the quantity and infectivity of HIV virions in a lymphoblastic cell line [65]. Surprisingly, this inhibition continued throughout three consecutive passages, without recovering viral titers [65]. Thus, the role of HSF1 in HIV infection is yet to be fully elucidated, as altogether there seem to exist some paradoxical effects for HSF1 during infection with this virus.

On the contrary, HSF1 may act as an innate repressor of inflammation induced by HIV [66]. Indeed, HSF1 overexpression inhibits HIV‐induced inflammatory responses in THP‐1 cells (a human monocytic cell line), and contrarily, HSF1 deficiency is associated with an increased inflammatory response [66]. This effect was due to a competition between HSF1 and nuclear factor‐κB (NF‐κB) in the nucleus, with HSF1 having an inhibitory effect over NF‐κB binding to the HIV long terminal repeats (LTRs), which leads to impaired transcription of viral genes and a reduced inflammatory response [66]. Thus, the inhibitory effect that HSF1 has over inflammatory responses could be further explored so that it could be used as a potential treatment for viral infections that induce inflammatory processes, such as SARS‐CoV‐2, the human papilloma virus, hepatitis C virus, and hepatitis B virus, among others.

A relation between HSF1 and the hepatitis B virus (HBV) has also been reported [67]. The p53‐binding protein 2 (apoptosis‐stimulating protein of p53‐2, ASPP2) can inhibit HBV‐induced hepatocyte autophagy in a p53‐independent manner [67]. Furthermore, the inhibition of autophagy in hepatocytes has been reported to inhibit HBV replication. Interestingly, ASPP2 binds to HSF1 in HBV‐infected cells, which in turn impedes its nuclear translocation. Importantly, the interaction between ASPP2 and HSF1 inhibits HSF1 nuclear translocation and inhibits the transactivation of the autophagy‐related protein 7 (Atg7), with an overall reduction in hepatocyte autophagy [67]. These findings indicate that, by regulating Atg7 transcription, HSF1 enables ASPP2 to reduce autophagy in hepatocytes and, therefore, inhibit HBV replication.

During vaccinia virus (VACV) infection, the host mRNA transcriptome is reshaped with several genes being downregulated [68]. According to this study, 611 host genes were upregulated upon VACV infection and this subset of genes was strongly enriched in genes that are regulated by HSF1 [68]. Additionally, HSF1 was also upregulated after VACV infection and was reported to be phosphorylated, translocate to the nucleus, and to increase the transcription of HSF1‐target genes [68]. Furthermore, the activation of this transcription factor supported viral replication and the inhibition of HSF1, as well as some targets of HSF1 such as HSP27, HSP70, and HSP90 blocked viral infection and replication [68], suggesting that HSF1, as well as HSF1‐induced proteins and their pharmacological regulation, could be potential treatments against VACV.

On the contrary, a cell line which overexpresses constitutively activated HSF1 (cHSF1) was found to induce an oncolytic effect in *in vitro* and *in vivo* studies, by promoting the replication of oncolytic adenovirus Adel55 [69]. Additionally, the overexpression of cHSF1 through its insertion into Adel55 (Adel55‐cHSF1) was found to induce a tumor‐specific immune response when used as a strategy for cancer gene therapy in immunocompetent hosts [69]. Consequently, Adel55‐cHSF1 induced a tumor‐specific immune response in mice with tumors [69].

Furthermore, a role for HSF1 in dengue virus (DENV) replication, both *in vitro* and *in vivo,* has also been assessed. HSF1 is activated during DENV infection in a Ca^+2^‐ and protein kinase A‐dependent manner [70]. Interestingly, the inhibition of HSF1 reduced DENV replication in THP‐1 cells and in primary human monocytes [70]. On the contrary, activated HSF1 induced DENV replication via the upregulation of Atg7, which is related to autophagy and is crucial for the replication of this virus [70]. The activation of HSF1 by heat stress also facilitated DENV replication, and in virus‐infected brains, the presence of activated HSF1 increased Atg7 and the induction of autophagy [70]. Consistently, the inhibition of HSF1 in this context resulted in reduced autophagy, as well as a reduction in viral protein expression, neuropathy, and mortality [70]. Therefore, the activation of HSF1 may be beneficial during DENV infections, and therefore, its inhibition may be a potential therapeutic strategy.

Additionally, increased temperature conditions have been reported to induce the transcription of the Epstein–Barr nuclear antigen 1 (EBNA1) in EBV‐transformed B95‐8 and LCL cell lines (a marmoset B cell line transformed by EBV and a EBV‐transformed human B cell line, respectively), which arose from the Q promoter (Qp)‐initiated transcripts [71]. This viral protein is consistently expressed in all malignancies associated with EBV, and it is reported to be crucial for the initiation of viral DNA replication, with Qp being the key promoter that regulates its expression [71]. Importantly, a high affinity and functional HSE was found in the Qp, and furthermore, HaCaT cells (a spontaneously transformed human keratinocyte cell line) co‐transfected with a plasmid encoding HSF1 and Qp‐luciferase displayed increased Qp activity [71]. Consistently, HSF1 gene silencing with interference RNA resulted in attenuated heat‐induced EBNA1 expression, indicating that EBNA1 expression is regulated by HSF1 [71]. Thus, it is likely that HSF1 may regulate the expression of EBNA1 through its binding to an HSE in the Qp promoter.

The human cytomegalovirus (HCMV) is able to expand the lifespan of monocytes through the stimulation of a non‐canonical Akt pathway after viral entry, which in turn leads to the increased expression of antiapoptotic proteins [72]. Interestingly, a relation between HCMV‐activated Akt and HSF1 has been described [72]. Activation of Akt during HCMV infection activates HSF1, which in turn upregulates the mTOR pathway that promotes the synthesis of cap‐ and internal ribosome entry site (IRES)‐containing mRNAs that encode antiapoptotic proteins [72]. Interestingly, the switch from cap‐dependent to IRES‐mediated translation usually occurs under conditions of cellular stress [72]. Thus, HCMV may benefit from HSF1 activation in order to induce the synthesis of certain proteins.

**Fig. 2:**
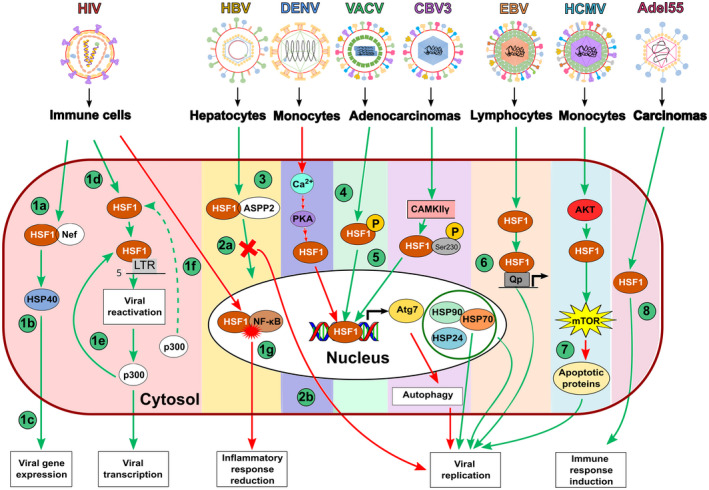
Schematic representations of the participation of HSF1 in viral infections. Red arrows indicate inhibitory pathways, while green arrows indicate activation pathways. From left to right: (1a) HSF1 associates with Nef, an early viral protein produced during HIV‐1 infection and (1b) activates HSP40, which promotes (1c) viral gene expression. (1d) HSF1 promotes the reactivation of HIV from latency, by binding to the 5'LTR in the viral genome and (1e) promotes the recruitment of protein complexes, such as p300. (1f) Additionally, HSF1 recruits p300 for self‐acetylation. (1g) HSF1 acts as a repressor in HIV‐induced inflammation, which occurs through a competition between HSF1 and nuclear factor κB (NF‐κB), which inhibits the NF‐κB pathway. (2a) HSF1 binds to ASPP2, which blocks the translocation of HSF1 to the nucleus and impairs Atg7 transcription, (2b) thus preventing autophagy and the replication of the hepatitis B virus (HBV) in hepatocytes. (3) HSF1 promotes autophagy through the transcription of Atg7 and inhibits dengue virus (DENV) replication. (4) HSF1 and heat shock proteins (HSPs), such as HSP90, HSP70, and other HSPs promote the replication of vaccinia virus (VACV). (5) Coxsackievirus B3 (CVB3) activates HSF1 and promotes the transcription of the gene of HSP70 through which downstream interactions promote viral replication. (6) There is a heat shock response element (HSE) in the viral genome of Epstein–Barr virus (EBV), specifically in the Qp gene. HSF1 binds to Qp promoting the initiation of viral replication in EBV‐infected cells. (7) The human cytomegalovirus (HCMV) promotes HSF1 activation to inhibit apoptosis, thus extending the lifespan of infected monocytes. (8) Finally, a constitutively active mutant of HSF1 (cHSF1) induces viral replication, and its overexpression induces a tumor‐specific immune response when using the oncolytic adenovirus Adel55.

The coxsackievirus B3 (CVB3) is known to exploit host cellular machineries during its replication cycle and to interact with host chaperones, such as HSP70 [73]. A cap‐independent translation of this protein has been described during viral infection, possibly due to an IRES within the mRNA of the HSP70 transcript [73]. Interestingly, upon CVB3 infection, the Ca^2+^/calmodulin‐dependent protein kinase γ (CaMKIIγ) has been reported to be activated, which leads to the activation of HSF1 due to the phosphorylation of a serine residue in position 230 of this protein, and the consequent enhancement of *HSP70* transcription [73]. Additionally, it has been reported that HSP70‐1 (a member of the HSP 70 protein family) stabilizes the CVB3 genome through its binding to an AU‐rich element (ARE) present in the 3′ untranslated region of the CVB3 RNA, which favors viral replication and enhances immune infiltration into the heart during the development of infection‐mediated myocarditis [73]. Therefore, activation of HSF1 and the consequent upregulation of HSP70 is beneficial for CVB3 [73]. How CVB3 infection leads to CaMKIIγ‐ and consequently HSF1 activation is yet unknown, but it is thought to be through a phosphorylation of a threonine residue (Thr286) in CaMKIIγ [73]. The participation of HSF1 in the replication cycle of the viruses discussed above is summarized in Fig. 2.

## Interrelationship between HSF1 and HSPs

The HSP family is composed of five subfamilies, which are classified according to their molecular weight, namely HSP60, HSP70, HSP90, HSP100, and a subfamily of small HSPs [74, 75]. The main drivers of the transcription of HSPs are HSFs [28]. Once HSF1 is oligomerized into its active homotrimer, it binds to the HSE of target genes, which leads to a rapid increase in the transcription of genes encoding proteins such as HSP90 and other chaperones such as HSP27, HSP40, and HSP70 [32, 76, 77].

Heat shock proteins play an important role in regulating the activity of HSF1. Under non‐stress conditions, HSF1 occurs in its monomeric form associated with molecular chaperones, mostly with HSP90 [27, 78]. Thus, in normal conditions HSFs are sequestered by molecular chaperones and these proteins form a multi‐molecular chaperone complex composed by HSPs, such as HSP40, HSP70, and HSP90, and other proteins such as 14‐3‐3 which contribute to the repression of this transcription factor [28, 29, 30]. When heat stress is present, HSP90 is released from HSF1, due to an increase in misfolded proteins that are sensed by the molecular chaperone, which allows HSF1 to form a homotrimer and its activation [29, 79].

A reduced expression of HSP90, but not of other HSR proteins, such as HSP70, heat‐shock organizing protein (Hop), and HSP40, induces the activation of HSF1, without inducing the transcription of HSP genes, and thus the inhibition of HSP90 is not the only factor needed to induce the transcription of HSPs [80, 81]. Interestingly, HSP90 has been shown to inhibit HSF1 activation and the binding of the latter to target DNA, whereas HSP70 inhibits the transactivation capacity of HSF1 [82]. Additionally, other studies have also reported that HSP40 and HSP70 inhibit the transactivation capacity of HSF1, likely due to the recruitment of the HSP70‐interacting transcriptional co‐repressor CoREST [82, 83]. However, the exact role that these chaperones play in HSF1 modulation is still controversial due to contradictory findings. For instance, the use of geldanamycin, an HSP90 inhibitor, results in HSF1 activation [80]. On the contrary, *in vitro* studies in which heat stress was applied, it was found that HSP90 induces HSF1 trimerization [27, 43]. Additionally, *in vivo* experiments show that the overexpression of HSP70 alone is not sufficient to suppress HSF1 DNA‐binding, but may play a role in the inactivation of this transcription factor after prolonged thermal stress [29, 30]. Interestingly, in human cells treated with sodium salicylate, HSF1 oligomerized and bound to the promoter of target genes such as *HSP70*, but its transcription was not induced [84]. Thus, oligomerization of HSF1 alone is not sufficient for promoting the transcriptional activity of this transcription factor.

Due to the diverse roles played by HSPs in viral infections (extensively reviewed by Wan et al. [32]), a close look into the factors promoting the expression of these proteins could provide further insights on the role of HSF1 in the replication cycle of viruses and the identification of this transcription factor as a potential target for antiviral treatments.

## Pharmacological modulation of HSF1

Heat shock factor 1 is being increasingly related to different pathologies, such as cancer and neurodegenerative diseases, and thus, interest is mounting on identifying drugs that modulate this transcription factor [85]. For instance, high levels of HSF1 correlate with poor prognosis in cancer patients [86, 87]. Additionally, HSF1 has been reported to drive oncogenesis by mediating the activation of genes that enable the initiation and maintenance of cancer cells through shifts in processes such as cell cycle control, metabolism, protein translation, and proliferation [88]. This has led to the assessment of different HSF1 inhibitors, such as the drug named NXP800, which is being tested in a phase I clinical trial. This drug has been shown to increase apoptosis of cancer cells in ovarian clear cell carcinoma (ClinicalTrials.gov Identifier: NCT05226507) [89]. Another HSF1 inhibitor is DTHIB, which has been extensively investigated and has been directly related to a reduction in the viability of prostate cancer cells, by decreasing the expression of antiapoptotic genes [90]. These drugs have not been tested in the context of viral infection, yet they may have effects that limit viral progression through the inhibition of HSF1.

Another drug that inhibits HSF1 with potential antiviral effects is KRIBB11. This drug binds directly to HSF1 and inhibits its interaction with target sequences in the DNA. KRIBB11 has been reported to significantly decrease the transcription of HSF1‐controlled genes, such as *HSP70, EGFR, MET,* and *AXL* and to promote the death of lung, glioblastoma, and myeloma cancer cells [91, 92, 93]. CCT261236 is a drug that decreases the activity of HSF1 and consequently the expression of HSPs [93]. Again, the potential antiviral activities of these drugs have been poorly assessed or not assessed at all. Two other inhibitors of HSF1 activity, and consequently HSP expression, are triptolide and KNK437, which promote the death of immortalized cells [94, 95]. For the latter, a pro‐apoptotic effect was seen through the downmodulation of the antiapoptotic proteins BCL2 and MCL1 in L363 cells [95].

On the contrary, drugs that promote HSF1 activity are also being evaluated as potential treatments, namely in the context of neurodegenerative diseases, such as Alzheimer's, Parkinson's disease, and amyotrophic lateral sclerosis [3]. One such drug is HSF1A, which positively modulates HSF1 as corroborated by an observed increase in HSP70 and HSP25 expression after treatment with this compound in a dose‐dependent manner [96]. Noteworthy, HSF1A specifically interacts with the TRiC/CCT complex and induces the activation of HSF1, which suggests a possible regulatory role for the TRiC/CCT complex over HSF1 [96].

Alternatively, due to the association between HSP90 or HSP70 and HSF1, some drugs aimed at positively modulating the activity of HSF1 target these chaperones, in order to release HSF1 so that it can exert its activity. One of these drugs, named geranylgeranylacetone (GGA) which targets HSP70 [97, 98, 99], while geldanamycin (17‐AAG) [100] and riluzole [101, 102] target HSP90. Whether these HSF1‐activating drugs may have antiviral effects remains unknown and thus should be determined experimentally to define if such an approach may have potential antiviral potential.

On the contrary, Celastrol activates HSF1 and leads to an increase in energy expenditure, increased insulin resistance, increased mitochondrial function in fat tissue and muscle cells, as well as hepatic steatosis in a high‐fat diet in 10T1/2 cells and primary fat SVF cells [103]. Regulation of energy expenditure is accomplished by the activation of PGC1α, a transcriptional coactivator that is a central inducer of mitochondrial biogenesis in cells and which modulates metabolic programing in adipose tissues and muscle [103]. The mechanisms of action of the different drugs targeting HSF1 and the pathologies in which they have been described are summarized in Table 1.

**Table 1:** Pharmacological modulation of HSF1.

Drug	Mechanism of action	Cell type in which its effects have been described	Related pathology	References
Drugs that inhibit or negatively modulate HSF1 activity				
NXP800	Inhibitor of the HSF1 pathway	Ovarian clear cell carcinoma	Cancer	[89]
DTHIB	Binds to the HSF1 DNA‐binding domain (DBD)	Human prostate cancer cell line (CRPC cell line C4‐2)	Cancer	[90]
KRIBB11	Binds to HSF1	Papillary Adenocarcinoma (NCI‐H820), Non‐small cell lung cancer (PC9‐ErlR), Glioblastoma (A172), Myeloma (KMS‐11), and Plasmacytoma (H929) cell lines	Cancer	[91, 92]
Triptolide	Inhibits the transactivation function of HSF1	Immunoglobulin A Lambda Myeloma (MM.1S) and multiple myeloma (INA‐6) cell lines	Cancer	[94]
CCT251236	Inhibits HSF1‐mediated HSP27 induction	Myeloma and plasmacytoma cell lines (KMS‐11 and H929, respectively)	Cancer	[93]
KNK437	Blocks HSF1‐mediated transcription	Plasma cell leukemia (L363) cell line	Cancer (multiple myeloma)	[95]
Drugs that activate or positively modulate HSF1 activity				
HSF1A	Negatively modulates the activity of the TRiC/CCT complex	Fibroblast (MEF) cell line	Neurodegenerative diseases	[96]
17‐AAG	Inhibits HSP90 by binding to its amino‐terminal	Lung carcinoma (A549) cells	Cancer	[100]
Riluzole	Unknown	Glioblastoma neuroprogenitor cells (NG108‐15)	Parkinson’s disease	[101, 102]
Geranylgeranylacetone	Induces the phosphorylation and nuclear translocation of heat shock factor 1 (HSF1)	Fibroblasts (CCD‐25SK) and OA cells, lung and cardiac tissue	Rheumatoid arthritis, lung injury/fibrosis, myocardial injury	[97, 98, 99]
Celastrol	Involved in PKC activation (translocation of PKCδ), which primes the phosphorylation of HSF1	Fibroblast sarcoma cells (10T1/2) and primary fat SVF cells	Obesity, insulin resistance	[103]

## Concluding remarks

Heat shock factor 1 is not only directly activated in response to increased temperature, but many other triggers also activate HSF1, or can modulate its threshold of activation. Importantly, several studies show that this transcription factor plays significant roles in the replication cycle of some viruses and that its involvement is independent of heat shock. The latter suggests that either viral determinants or host factors modulated by viral infection are impacting directly on HSF1 or on factors that regulate its function. Given that virus‐infected cells may undergo some level of UPR due to sustained viral protein translation during infection, it is also possible that a link may exist between this response and HSF1 [7, 8, 104]. Given that a negative feedback loop between HSPs and HSF1 allows the regulation of HSF1 function, it will be of interest to assess this potential relationship in depth in the context of viral infections that interact with HSPs or induce their expression and to determine what is the contribution of HSF1 activation during the replication cycle of different viruses.

Additionally, it will also be important to evaluate the effect of varying levels of HSF1 expression in different cell types on the modulation of this transcription factor over viral infections, as HSF1 is not equally expressed among cell types and its expression will differ depending on environmental and cellular conditions [44]. Also, it will be beneficial for future research to explore whether a relation between viral infections and other HSF members exists, given the similarities and differences between the transcription factors in this family.

Because multiple drugs that positively or negatively modulate HSF1 activity are currently being tested in clinical trials for cancer and neurodegenerative diseases, it will be interesting to evaluate whether these drugs have positive or negative effects for the host in the context of viral infections, potentially serving as novel strategies to counteract viral replication in the infected individual. To our knowledge, to date there are no reports describing the use of such drugs to target HSF1 in the context of viral infections.

Although there is very little information regarding the effect that the modulation of HSF1 activity may have over viral infections, there are multiple connections between gene products associated with this transcription factor and viral infections. Thus, we foresee that targeting HSF1 will be an interesting new approach for the treatment of viral infections, given the constant need for identifying and developing new drugs to combat this type of pathogens. Yet, it is important to note that altogether it will be necessary to corroborate that modulating HSF1 in the host will not harm the individual, particularly in scenarios in which there are significant increases in body temperature, such as during fever, as in this case altering the function of HSF1 could be deleterious for the host’s tissues.

## Conflict of interest

The authors declare no conflict of interest.

## Author contributions

All authors wrote and reviewed the manuscript. AN, BD, and PG designed the figures.

## Data Availability

The data that supports this review is available and was found in PubMed at https://pubmed.ncbi.nlm.nih.gov.
